# The Ultrasound-Assisted Extraction of Polyphenols from Mexican Firecracker (*Hamelia patens* Jacq.): Evaluation of Bioactivities and Identification of Phytochemicals by HPLC-ESI-MS

**DOI:** 10.3390/molecules27248845

**Published:** 2022-12-13

**Authors:** María del Carmen Gutiérrez-Sánchez, Pedro Aguilar-Zárate, Mariela Ramona Michel-Michel, Juan Alberto Ascacio-Valdés, Abigail Reyes-Munguía

**Affiliations:** 1 Facultad de Estudios Profesionales Zona Huasteca, Universidad Autónoma de San Luis Potosí, Maestría en Ciencias Bioquímicas, Romualdo del Campo # 501 Fracc, Rafael Curiel, Ciudad Valles 79060, San Luis Potosí, Mexico; 2 Departamento de Ingenierías, Instituto Tecnológico de Ciudad Valles, Tecnológico Nacional de México, Carretera al Ingenio Plan de Ayala Km. 2, Col. Vista Hermosa, Ciudad Valles 79010, San Luis Potosí, Mexico; 3 Food Research Department, School of Chemistry, Autonomous University of Coahuila, Blvd. V. Carranza e Ing, José Cárdenas Valdez S/N, Saltillo 25280, Coahuila, Mexico

**Keywords:** flavonoids, hydroxycinnamic acid, coumarins, antioxidants, antimicrobial, Taguchi methodology

## Abstract

The objective of the present work was to optimize the extraction of phytochemicals from *Hamelia patens* Jacq. by ultrasound-assisted extraction. Taguchi L9 orthogonal array was used to evaluate the factors solid/liquid ratio (1:8, 1:12, and 1:16), extraction time (10, 20, and 30 min), and ethanol concentration (0, 35, and 70%). Total polyphenols were the response variable. Chromatographic fractionation using Amberlite XAD-16 was carried out and the total polyphenols, flavonoids, and condensed tannins were quantified. The redox potential, the reduction of the 2,2-diphenyl-1-picrylhydrazyl (DPPH), and the lipid oxidation inhibition were determined. Anti-bacterial activity was evaluated. The phytochemicals were identified by liquid chromatography coupled to mass spectrometry. Optimal extraction conditions were a solid/liquid ratio of 1:16, ethanol of 35%, and 10 min of ultrasound-assisted extraction. Maximum polyphenol content in the crude extract was 1689.976 ± 86.430 mg of gallic acid equivalents (GAE)/100 g of dried plant material. The purified fraction showed a total polyphenols content of 3552.84 ± 7.25 mg of GAE, flavonoids 1316.17 ± 0.27 mg of catechin equivalents, and condensed tannins 1694.87 ± 22.21 mg of procyanidin B1 equivalents, all per 100 g of purified fraction. Its redox potential was 553.93 ± 1.22 mV, reducing 63.08 ± 0.42% of DPPH radical and inhibiting 77.78 ± 2.78% of lipid oxidation. The polyphenols demonstrated antibacterial activity against *Escherichia coli, Klebsiella pneumonia*, and *Enterococcus faecalis*. The HPLC-ESI-MS analysis revealed the presence of coumarins, hydroxycinnamic acids, and flavonoids.

## 1. Introduction

In recent years, the food, cosmetic and pharmaceutical industries have commonly performed extractions of bioactive compounds obtained from natural sources [1,2]. *Hamelia patens* Jacq. (Rubiaceae) has been reported to possess compounds with important bioactivities, because it has been used in traditional Mexican medicine [3,4,5]. It has been reported to possess several bioactivities in vitro and in vivo, including antihemorrhagic [6], hepatoprotective, antioxidant [7], antihyperglycemic [8], and antibacterial [5] properties, among others. The tested extracts contained phytomolecules such as epicatechin, chlorogenic acid [8], rutin, isoquercetin and soyasaponin Bb, which were first identified in the leaves of the plant [9]. However, the obtention of bioactive compounds depends mostly on the extraction method and the solvent used.

The extraction procedures of bioactive compounds commonly involve the use of organic solvents, steam distillation, high hydrostatic pressures, or countercurrent extraction. The main disadvantages of these procedures are their requirements, including large amounts of organic solvents, energy, time, and the low yields sometimes obtained [10,11]. In recent years, the usage of clean extraction technologies, including ultrasound-assisted extraction, supercritical fluid extraction, and accelerated microwave extraction, has been widely promoted. Ultrasound-assisted extraction (UAE) is the least expensive and most efficient method for the obtention of bioactive compounds, such as aromatic, essential oils, polyphenols, isoflavonoids, saponins, pigments, and sugars. The UAE must be used when the elevated temperatures of conventional procedures affect the stability of the active components [12,13]. The UAE is an example of an alternative extraction technique that offers many advantages, including high efficiency and productivity, low energy expenditure, and a reduction in solvent consumption, among others [14,15,16].

To select the appropriate conditions for the extraction of compounds, bioactive statistical strategies have been employed [14,17,18]. The Taguchi methodology is a robust optimization strategy focused on the use of many simultaneous parameters and small experimental trials [19,20]. The methodology includes the usage of orthogonal arrays that allow cost and time reduction [21]. In addition, the robust design improves the quality of the tested processes due to the versatile analyzing models. For that reason, the methodology is considered better than factorial and fractional factorial designs due to the extent of information obtained from the analysis of very few experimental runs [12].

The objective of the present work has been to optimize the extraction of phytochemicals from *H. patens* leaves by using the ultrasound-assisted extraction method and the Taguchi methodology. Likewise, biological activities (antioxidant and antimicrobial) and the characterization of phytochemicals by high-resolution liquid chromatography coupled to mass spectrometry with electrospray ionization (HPLC-ESI-MS) were performed.

## 2. Results

### 2.1. Optimization of Polyphenols Extraction

The Taguchi methodology is a robust way for finding the optimal extraction conditions. Table 1 shows the experimental results by using the L9 (3^3^) orthogonal array. The conditions from treatment number 7 and 9 resulted in a good polyphenols extraction yield (1636.80 ± 79.20 and 1652.33 ± 1.33 GAE mg/100 mg, respectively). The higher yield was obtained in treatment 9. All the results were expressed as gallic acid equivalents per 100 g of dried plant material. It is worth mentioning that the content of total polyphenols was the phytochemical of interest for this study.

The experimental data presented in Table 1 was considered to carry out the statistical analysis using the analysis mode of higher the better from the Taguchi methodology. The analysis of variance (ANOVA) shows the influence of every factor on the process (Table 2). In the present work, the most important factor was the solid/liquid ratio (54.09%), followed by the ethanol concentration (17.09%). It indicates that the amount of vegetal material and the solvent concentration must be taken into consideration for the extraction process of polyphenols from *H. patens*.

**Figure 1:**
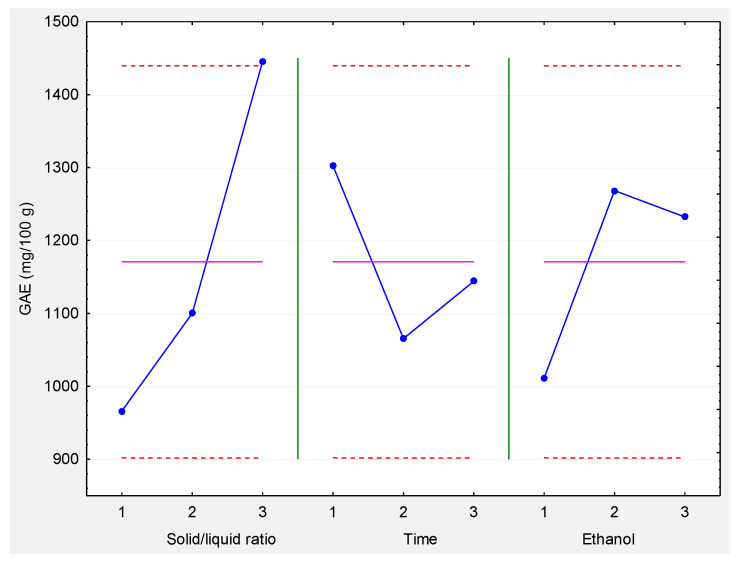
Individual factors performance at different levels. The continuous line represents the mean of all the treatments (1170.70 GAE mg/100 g of dried plant material) and the red dashed lines are the ±2 standard error of all the treatments.

Figure 1 shows the performance of the three factors at their different levels. The solid/liquid ratio performance indicates that by increasing the ratio, a higher response is obtained. On the contrary, the time of extraction has a better performance at the lowest level. The ethanol concentration showed a quadratic effect, indicating that the optimal concentration for the best extraction of polyphenols is 35%.

The optimal extraction conditions for the polyphenols from *H. patens* are shown in Table 3. According to the software Statistica 7, a solid/liquid ratio of 1:16, a sonication time of 10 min, and 35% of ethanol predicts the extraction of 1675.23 mg of polyphenols. The experimental validation improved the expected result from the software. It resulted in the extraction of 1689.98 ± 86.430 GAE mg/100 g of dried material.

### 2.2. Characterization of Phytochemicals Extracted from H. patens

The extracted polyphenols from *H. patens* were partially purified by open-column chromatography using Amberlite XAD-16 as the stationary phase. The ethanolic fraction containing the extracted phytochemicals was recovered. A recovery yield of 1.7% of dried fractionated phytochemicals from *H. patens* leaves was obtained.

A screening of the main chemical groups of the phytochemicals extracted from *H. patens* was carried out. In this qualitative test, the presence of alkaloids, sterols, flavonoids coumarins, and phenolic hydroxyls was found. However, the presence of unsaturation groups, sesquiterpenolactones, saponins, and cardiotonic glucosides was not found.

Table 4 shows the quantitative characterization of the phenolic content and antioxidant activities of the phytochemicals extracted per 100 g of purified polyphenol fraction. A total polyphenols content of 3552.84 ± 7.25 was found, expressed as gallic acid equivalents of milligrams per 100 g of purified polyphenol fraction. The flavonoids content and the condensed tannins were evaluated as catechin (CatE) and procyanidin B1 (PC-B1E) equivalents, respectively. The total phenolic acids content shows interesting results regarding their value as antioxidative agents. They have important redox potential (553.93 ± 1.22 mV), DPPH radical reduction (63.08 ± 0.42%), and lipid oxidation inhibition (77.78 ± 2.78%).

Despite the good antioxidant activity of *H. patens* polyphenols, the inhibition of Gram-negative and Gram-positive bacterial strains resulted in null for most of the tested strains (Table 5). Both Gram-positive and Gram-negative bacteria were selected for the development of the study: *Pseudomonas aureuginosa* ATCC 10145, *Enterobacter cloacae* (Clinical isolate), *Escherichia coli* ATCC 15597, *Escherichia coli β-lactamase negative*, *Klebisella pneumoniae*, *Staphylococcus aureus* ATCC 29213, *Staphylococcus epidermidis* and *Enterococcus faecalis* ATCC 19433. Most of the strains were inhibited by the control treatment, except *E. cloacae* (Clinical isolate) and *S. aureus ATCC 29213*. However, reliable results were obtained against the Gram-negative *E. coli β-lactamase negative*, *K. pneumoniae* and the Gram-positive *E. faecalis*, reaching inhibitions of 90.5, 75.0 and 80.0%, respectively. Furthermore, low amounts of total polyphenols were found to be minimum inhibitory concentrations for the three later strains.

### 2.3. HPLC-ESI-MS Characterization

**Figure 2:**
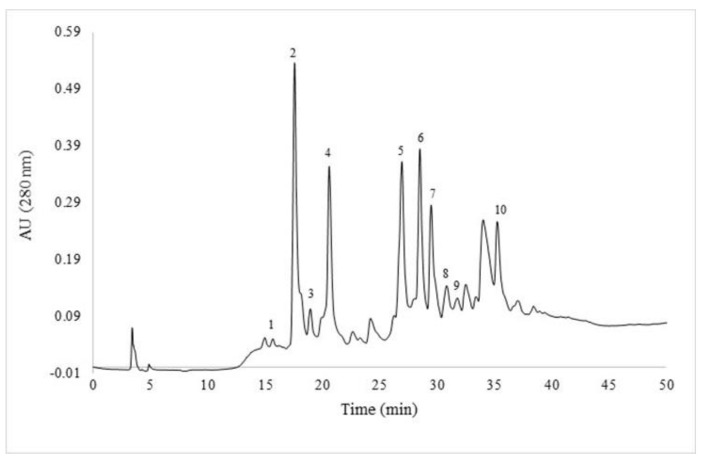
HPLC chromatogram showing the identified phytochemicals from *H. patens*.

The purified polyphenol fraction extracted from *H. patens* was submitted for an HPLC-ESI-MS/MS analysis. The results showed the presence of 10 ionized compounds (Table 6, Figure 2 and Figure S1). The tentative identification of the compounds was based on the ionization of the masses and their fragmentation pattern. In addition, the results were compared against data previously reported in the literature. The presence of hydroxycinnamic acids, coumarins, an alkaloid, and flavonoids (flavanols, flavones, and flavonols) in both glycosylated and aglycone forms was identified.

## 3. Discussion

### 3.1. Total Polyphenols Content

The obtained data in all conditions (total polyphenols expressed as mg of GAE/100 g) were analyzed in terms of the higher the better, due to the interest in obtaining the highest concentration of polyphenols in the shortest time with the least amount of solvent. The solid/liquid ratio had the higher percentage of contribution (54.09%), indicating that this factor is the most important in the extraction process of polyphenols from *H. patens*. Socas-Rodríguez et al. [31] propound that ethanol is the best option for the extraction of bioactive compounds in the food industry, specifically phenolic compounds, since it is biologically safe, is related to these compounds, and is also considered a GRAS (generally recognized as safe) product.

To validate the optimal extraction condition, the extract was prepared in triplicate and the total polyphenol content was determined for each extract. The predicted value was 1675.23 GAE mg/100 g, while the experimental validation result was 1689.98 ± 86.43 GAE mg/100 g. Fontanills et al. [5] reported 212.73 mg of polyphenols per g of dry material in methanolic extracts of *H. patens*. They mentioned that the use of methanol instead of ethanol enhances the extraction of phytochemicals. However, they carried out the extraction by maceration. Another study carried out by Giraldo Vásquez and Ramírez Aristizabal [32] in methanolic extracts of *Palicourea guianensis*, known as “cafecillo” in the Rubiaceae family, reported 1047 GAE mg/100 g. Although the authors used the ultrasound-assisted extraction method, large amounts of methanol (100 g of material in 1 L of methanol, six successive extractions of 20 min) were used. Therefore, the results obtained in the present work demonstrate the efficiency of the process for the extraction of phytochemicals in the leaves of *H*. *patens*; since this process was carried out in a short period, a very low concentration of organic solvent was required.

### 3.2. Characterization of Extracted Bioactive Compounds

#### 3.2.1. Chemical Identification and Antioxidant Properties

In the purified phytochemicals fraction of *H*. *patens*, the chemical groups of alkaloids, sterols, flavonoids, coumarins, and phenolic hydroxyls were identified. These results are largely in agreement with those reported by Noor et al. [4] and Rubio Fontanills et al. [5], who report the presence of alkaloids, tannins, glycosides, saponins, steroids, flavonoids, terpenoids, and coumarins in ethanolic extract.

The content of total polyphenols, flavonoids, and condensed tannins in the purified phytochemicals fraction of *H. patens* was determined. All studies were performed in triplicate, obtaining an average total polyphenol content of 3552.84 ± 7.25 mg of gallic acid equivalents per 100 g of purified *H. patens* phytochemicals. Regarding flavonoid content, an average of 1316.17 ± 0.27 mg of catechin equivalents/100 g of the purified phytochemicals fraction of *H. patens* was obtained. This result differs from that reported by Rugeiro-Escalona et al. [8], who reported 399 mg of quercetin/100 g of extract. This difference may be due to uncontrolled external factors, such as the time of leaf collection, soil type or climatic conditions, as well as the method used for the determination, the type of solvent, and the standard utilized. In the determination of condensed tannins, an average of 1694.87 ± 22.21 mg PC-1/100 g of the purified phytochemicals fraction of *H. patens* was obtained. These results also differ from those reported by Rugeiro-Escalona et al. [8]. They reported 291.3 mg of catechin equivalents/g of phytochemicals purified from *H. patens.* It is worth mentioning that the antioxidant activity of the purified phytochemicals fraction of *H. patens* may be mainly due to the presence of procyanidins, since according to Vázquez-Flores et al. [33], the antioxidant property of condensed tannins in vitro and in vivo indicates that they are effective free radical scavengers, which prevent tissue oxidation better than vitamin C, vitamin E, and β-carotene. The results obtained from the purified phytochemicals fraction confirm that *H. patens* is an excellent source of phenolic compounds such as phenolic acids and flavonoids; these are the main antioxidant properties.

Table 4 shows the redox potential, percentage reduction of the DPPH radical, IC50, and the percentage inhibition of lipid oxidation, respectively. In the determination of the redox potential of the purified phytochemicals fraction of *H. patens*, a value of 553.93 ± 1.22 mV was obtained, which means that the purified phytochemicals fraction of *H. patens* has good antioxidant activity. According to the results obtained by Van Dijk et al. [34], excellent antioxidant activity is considered to be when oxidation potentials below 600 mV are obtained. Reyes et al. [35] reported high antioxidant activity with redox potentials below 500 mV in infusions of *Spondias purpurea* L. (yellow plum). In a study by Muedas-Taipe et al. [36], in ethanolic extracts of *Bauhinia guianensis* var. kuntiana Aubl, they concluded that at low oxidation potentials (140 to 150 mV), the polyphenols showed good antioxidant activity. The DPPH radical scavenging showed 63.08 ± 0.42% of free radical reduction. Sánchez Balcázar and Tanta-Flores [37] reported an 81.70% antioxidant capacity by the DPPH radical method in the ethanolic extract of blueberries. On the contrary, Peláez-Gutiérrez [38] suggests that the fractions obtained from the extract *Palicourea guianensis* Aubl. from the *Rubiaceae* family are below 50% reduction, which reveals that they did not present good antiradical capacity. Regarding the IC50, it was found that 67.49 mg/L of the purified phytochemicals fraction of *H. patens* can reduce the 50% of the DPPH radical. Kaushik and Singh [39] reported a concentration of ethanolic extract of *H. patens* of 116 mg/L to reduce 50% of the DPPH radical. Pacheco Coello et al. [40] report that artisanal green tea requires a concentration of 160.3 mg/L to reduce 50% of the DPPH radical.

Finally, it was found that the purified phytochemicals fraction of *H. patens* presents the ability to inhibit 77.78 ± 2.78% of lipid oxidation, a very similar result to that reported for the ethanolic extract of *Laurus nobilis* (76.86%) [17]. Furthermore, comparing the outcome with the ascorbic acid control (92% of inhibition), the application of *H. patens* polyphenols can be focused to inhibit the oxidation of oily systems [41]. This property can be attributed to the presence of flavonoids, such as catechin and quercetin, in the purified phytochemicals fraction of *H. patens*.

#### 3.2.2. Antibacterial Activity of the Purified Phytochemicals Fraction of *H. patens*

As was previously mentioned, the purified polyphenols from *H*. *patens* only showed an inhibitory effect on three of the bacteria studied: β-lactamase negative *Escherichia coli*, *Klebsiella pneumoniae,* and *Enterococcus faecalis*. These results differ from those reported by Rubio Fontanills et al. [5] and by Wong-Paz et al. [42], who observed inhibition with *H. patens* extract of *Staphylococcus aureus* and *Staphylococcus epidermidis, Salmonella typhi*, *Salmonella paratyphi*, and *Escherichia coli*. This may be attributed to the solvent used to solubilize the tested compounds, since 70% dimethyl sulfoxide was used in this study and not 70% ethanol as the other studies used. Ethanol has been reported to have an antimicrobial effect [43,44].

Regarding the minimum inhibitory concentration of the studied bacteria, it was found that for the inhibition of *K. pneumoniae*, the highest concentration of the purified phytochemicals fraction was required (250 µg/mL). Meanwhile, the lowest concentration (94 µg/mL) was used on *E. coli* β-lactamase negative. Vargas-Sánchez et al. [45] reported that the inhibition of the enzyme DNA gyrase, a fundamental enzyme for the process of transcription and DNA replication in bacteria, such as *E. coli* [46], is due to the action of quercetin and apigenin, compounds present in the phytochemicals purified from *H. patens*. Additionally, Pájaro et al. [47] reported that the flavonoids contained in the ethanolic extract of the petiole of *Rheum rhabarbarum* (rhubarb) have antibacterial activity against *K. pneumoniae*. The ability of the purified phytochemicals fraction of *H. patens* to inhibit bacterial growth was demonstrated using concentrations from 94 to 250 µg/mL, which were attributed to the presence of detected phenolic compounds such as quercetin, apigenin and coumarins.

### 3.3. HPLC-ESI-MS Analysis

Once the different chemical groups present in *H. patens* were screened, they were identified by HPLC-ESI-MS analysis. Previous studies carried out by our research group have demonstrated the presence of compounds such as Kaempherol-3-O-rutinoside, procyanidin B2, and epicatechin in *H. patens* extracts obtained by Soxhlet, percolation, and maceration [40]. Ten phenolic compounds were tentatively detected in the purified phytochemicals fraction of *H. patens* (Table 6).

The identification of the ionized compounds was based on the search for the main molecular ions and some of the fragments obtained from databases and scientific articles. In the chromatogram (Figure 2), peaks 1 and 2 represent the isomers of a coumarin, Scopoletin-7-O-glucoside, both peaks present a precursor *m*/*z* of 352.9 and its fragment *m*/*z* 191.05, which led to its tentative identification according to that reported by Rigane et al. [22] who first identified it in leaves of *Calendula officinalis* L. cultivated in Tunisia (North Africa). Yerlikaya et al. [48] reported a compound of *m*/*z* 355 ionized in positive mode as Scopoletin-7-O-glucoside in *Coronilla varia* L. extracts; this *m*/*z* also agrees with coumarin found in the purified phytochemicals fraction of *H. patens*. Erb et al. [49] report that coumarins present bactericidal and fungicidal activity, as well as several pharmacological properties that could help in the treatment of diseases such as cancer, Alzheimer’s or acquired immunodeficiency syndrome.

The ion found with *m*/*z* 576.9 (peak 3) corresponds to two Epicatechin units confirmed with a fragment of *m*/*z* 407.04 [21]. Peak 4 at *m*/*z* 288.9 was identified as (Epi)-Catechin according to its 245.96 fragmentation pattern [24]. The compounds in peaks 5 and **7** represent the same parent ion with *m*/*z* 593. Mass spectrometry data showed ion fragments of *m*/*z* 383.01 and *m*/*z* 353.12, which are characteristic of di-C-glycosylflavone fragmentations [26]. This indicates the presence of apigenin as an aglycone and two hexose residues. Therefore, the compounds were identified as the isomers of 6,8-di-C-glycosylapigenin, also known as Vicenin II [25,50]. These flavonoids, specifically of the flavone type, could bestow anti-inflammatory and healing properties on the plant [51].

A glycosylated flavonoid with *m*/*z* of 609 was tentatively identified as Quercetin-deoxyhexosyl-hexoside (peak 6), where the ion fragment with *m*/*z* 301.11 contributed to its identification. Di Majo et al. [52] argue that this type of flavonols contribute to reducing the risk of cardiovascular diseases, and they are also good antioxidants and antimicrobial agents. Vicente et al. [53] found that the chemical structure of quercetin confers antioxidant properties, since it acts as a protector against oxidizing species; in addition the consumption of quercetin in the diet is attributed to anticarcinogenic properties. Vargas-Sánchez et al. [45] pointed out that flavonoids such as quercetin and apigenin act by inhibiting the DNA gyrase of some Gram-positive and Gram-negative bacteria.

Peak 8 showed an ion of *m*/*z* 593 and it was tentatively identified as Kaempferol-3-*O*-rutinoside, the fragmentation gave the aglycone anion of *m*/*z* 285.1 [27,54]. This flavonol-type flavonoid contributes to the reduction of cardiovascular diseases [55].

According to the database *ReSpect for Phytochemicals* [29] and Hernández et al. [28], the parent ion with *m*/*z* 395 may correspond to an alkaloid (peak 9). However, the information could not be corroborated since no fragments were reported for the MS^2^ analysis; for that reason, it was considered an unknown compound. Regarding peak 10, it represents a precursor ion of *m*/*z* 340.9, and the database *ReSpect for Phytochemicals* [29] indicates that it is a phenylpropanoid. Among the phenylpropanoids, there are compounds such as trans-cinnamic acid, p-coumaric acid, its derivatives (caffeic acid), and coumarins. Olennikov et al. [30] identified a similar precursor ion indicating that it could be caffeic acid-*O*-glucoside. However, this could not be verified since the fragmentation pattern did not coincide with that reported by the aforementioned author. The results of the HPLC-ESI-MS^2^ analysis indicated that *H. patens* is a rich source of phenolic compounds that exhibit anti-inflammatory, wound healing, antioxidant and antimicrobial properties, which could be used in foods and/or drugs that would have the ability to reduce oxidative stress.

## 4. Materials and Methods

### 4.1. Plant Material and Extracts

Leaves of *H. patens* (Voucher HPHP-SP-102, deposited at Centro de Investigación Reserva de la Biosfera Mapimi) were collected from domestic yards in Ciudad Valles, S.L.P. in Mexico (Latitude: 21°59′41″ North, Longitude: 99°0′39″ West) during the months of June and July 2020. The leaves of *H. patens* were dried in a conventional oven (Linderberg/Blue, USA), at 55 °C for 48 h, until they reached a constant weight. The samples were pulverized with a blender (Osterizer, Mexico) to a particle size of 0.8 mm and stored at room temperature until use. The extracts were obtained by ultrasound in an ultrasonic bath (model 2510, BRANSON, CT, USA) using different extraction variables, according to Taguchi’s experimental design.

### 4.2. Optimization of Extraction Conditions

For selecting the better extraction conditions of bioactive compounds from *H*. *patens* leaves, three factors with three levels each were tested (Table 7).

The studied conditions were generated according to the Taguchi robust design, originated by using the *Statistica 7* program (Statsoft, Tulsa, OK, USA). The experimental matrix was an L9 (3^3^) orthogonal array showing nine experimental runs derived from the three factors and the three levels (Table 7) [12]. The extraction was performed at room temperature (25 °C) using the dried milled material (0.8 mm particle size) under the conditions depicted in Table 1.

### 4.3. Quantification of Total Polyphenols Content

The crude extracts obtained in each trial were assayed for quantifying the total polyphenols content according to the method referred to by Nossa González et al. [56] as follows: 125 μL of sample, 500 μL of distilled water and 125 μL of Folin–Ciocalteu reagent were added in a test tube. The solution was left for 6 min at room temperature to react. Then, 1.25 mL of Na_2_CO_3_ solution (7%) and 1 mL of distilled water were added. The mixture was left to react at room temperature (25 °C) for 90 min. The reaction was monitored at 760 nm in a UV-vis spectrophotometer (Genesis 10, Thermo Scientific, Waltham, MA, USA). The gallic acid standard curve (0–100 μg/mL) was used as a reference [57]. The results were expressed in GAE/100 g of dried plant material.

### 4.4. Optimization of the Extraction Conditions

The obtained results from the experimental matrix (Table 1) were entered into *Statistica 7* (Statsoft, Tulsa, OK, USA) to obtain the optimum extraction conditions. The function the higher the better was selected from the three categories (the lower the better, the higher the better, and the nominal the best) of quality characteristics in Taguchi methodology. It is expressed as the loss function L(*y*) = *k* × (1/*y*^2^) and represented as follows:

(1) E[L(y)]=−10∗Log10[(1n)∗∑(1yi2 )]

The factor −10 ensures that this ratio measures the inverse of “bad quality” and *n* represents the number of samples.

For significant factors, the percentages of their contributions were determined from Equation (1).

(2) $$P=\frac{SS_{i}}{SS_{T}}*100\%=\frac{SS_{i}-MS_{i}*df_{i}}{SS_{T}}*100\%$$

where *SS_i_* (square sum of *i* factor), *SS_T_* (total square sum), *MS_i_* (mean square of *i* factor), and *df_i_* (degrees of freedom of *i* factor) were obtained from the ANOVA.

To validate the result obtained in the previous step, the extract was obtained in triplicate using the optimal conditions predicted by the software, and total polyphenols were quantified.

### 4.5. Concentration of Total Polyphenols by Open-Column Chromatography

Once the optimal extraction conditions were defined, the polyphenols were concentrated using an open chromatographic column (25 mm × 1000 mm) packed with Amberlite XAD-16N (Sigma-Aldrich, St. Louis, MO, USA). The separation and purification of the bioactive compounds were carried out using the methodology of De la Rosa Hernández [58]. Firstly, an elution with water was carried out to remove water-soluble compounds, and then an elution with 70% ethanol was performed to recover a fraction rich in bioactive compounds. The aqueous fraction was discarded and the ethanolic fraction was recovered and concentrated in a rotavapor. The purified ethanolic fraction obtained was called the purified polyphenol fraction, and it was stored at 4 °C until use. It is worth mentioning that only the extract obtained under the expected extraction conditions (previous point) was concentrated and purified, and only a single fraction of purified polyphenols was obtained; this was characterized and its biological activity was measured.

### 4.6. Characterization of Bioactive Compounds

To characterize the bioactive compounds present in the purified phytochemicals fraction of *H. patens*, phytochemical screening was performed [59]. In addition, the quantification of total polyphenols [56], flavonoids [60], and condensed tannins [61] was determined. The results were expressed in mg GAE, mg CatE, and mg PC-B1E per 100 g of the purified phytochemicals fraction of *H. patens*, respectively. Furthermore, a phytochemical sieve was performed using the methodology of Chigodi et al. [59] to qualitatively identify the chemical groups found in the fractionated phytochemicals.

#### Antioxidant Activities

The antioxidant and antimicrobial properties of the purified phytochemicals fraction of *H. patens* were tested. The antioxidant properties were evaluated by measuring the redox potential [62], the reduction of the 2,2-diphenyl-1picrylhydrazyl radical (DPPH) [12], and the lipid oxidation inhibition [63]. The antimicrobial properties were evaluated via analyzing the inhibitory effect and the minimum inhibitory concentration (MIC) [64].

For both the characterization and the determination of antioxidant activity, a solution of the purified phytochemicals fraction of *H. patens* at a concentration of 1 mg/mL was prepared.

### 4.7. Identification of Phytochemicals by HPLC-ESI-MS^2^

The obtained extracts for validation were analyzed via Reverse Phase High-Resolution Liquid Chromatography, using a similar method to that reported by Aguilar-Zárate et al. [65]. It employed multi-module equipment that includes a ternary pump (Varian ProStar 230I, USA), autosampler (Varian ProStar 410, USA), and photodiode array detector (PDA) (Varian ProStar 330, USA). Ten micro-liters of every sample were submitted to a Denali C18 column (150 mm × 4.6 mm, 3.1 µm, Grace, USA) that was placed into an oven adjusted to 30 °C. Solvent A was acetic acid 3% and solvent B was acetonitrile. The flow rate was kept at 0.2 mL/min and the PDA was adjusted to 280 nm for the monitoring of phenolic compounds. The applied gradient was as follows: initial, 3% B; 5–15 min, 16% B linear; 15–45 min, 50% B linear. The column was then washed and reconditioned. Data were processed using the software Workstation Multi-Instrument (Version 6.2).

Tandem analysis, from HPLC to mass spectrometry, was carried out. The liquid chromatograph mass spectrometer was equipped with an electrospray source (Varian 500-MS, USA). All the samples were ionized in negative mode [M-H]^-^, and nitrogen, and helium were used as nebulizing and damping gases. The configuration of the ion source was a spray voltage of 5.0 kV, capillary voltage of 90 V, and temperature of 350 °C. Data collection and the process were performed by MS Workstation software (version 6.9) in the *m*/*z* range 50–2000. Selected precursor ions were fragmented in the order of MS^2^ [61]. The collision energy was fixed at 30 eV.

### 4.8. Inhibitory Effect and Minimum Inhibitory Concentration (MIC)

To study the antimicrobial activity of the purified phytochemical fraction of *H. patens*, the inhibitory effect was measured based on the technique described by Sánchez-Recillas et al. [64], with certain adaptations. Both Gram-positive and Gram-negative bacteria (*Pseudomona aureuginosa*, *Enterobacter cloacae*, *Escherichia coli*, *Escherichia coli* β-lactamase negative, *Klebisella pneumoniae*, *Staphylococcus aureus*, *Staphylococcus epidermidis* and *Enterococcus faecalis*) were selected for the development of the study. This type of bacteria produces major infections in the human organism and is indicative of contamination in food.

To determine the MIC of the purified polyphenol fraction of *H. patens* for bacteria that presented inhibition, the microdilution method described by Ramírez and Marin-Castaño [66] was used with some modifications, using a plate of 96 wells (12 mm × 8 mm).

### 4.9. Statistical Analysis

The results of the total phenolic content were analyzed using the software Statistica 7 (Statsoft, Tulsa, OK, USA). The analysis of variance (ANOVA) was determined based on experimental results. The expected optimum condition shown by the software was experimentally validated and the bias of the predicted response from the actual data was calculated. All the experimental trials were performed in triplicate. All the data were shown as the mean ± standard deviation.

## 5. Conclusions

The ultrasound-assisted extraction, combined with the Taguchi methodology, is efficient tool for the optimization of the extraction of phytochemicals from *H. patens* leaves. The purified polyphenols had both antioxidant and antimicrobial activities. These biological properties must be attributed to the presence of phenolic acids and flavonoids identified by HPLC-ESI-MS^2^. The detected phytochemicals were glucocafeic acid, scopolentin-7-*O*-glucoside, flavonoids, flavanols, such as epicatechin, flavones, such as apigenin adducts, flavonols, such as kaempferol and quercetin glycosides, and an alkaloid. These largely justify the antimicrobial and antioxidant properties of *H. patens* leaves. The present work describes the phytochemicals found in *H. patens*, proposing its ethnobotanical uses in therapeutic agents, food preservatives, and antioxidants.

## Figures and Tables

**Table 1:** Experimental matrix for the orthogonal array L9 (3^3^) and total content of polyphenols for the experimental treatments.

Run	Solid/Liquid Ratio (*w/v*)	Time (min)	Ethanol (%)	Total Content of Polyphenols (GAE mg/100 g) *
1	1:8	10	0	1074.16 ± 75.87
2	1:8	20	35	956.28 ± 61.56
3	1:8	30	70	868.05 ± 31.28
4	1:12	10	35	1196.54 ± 27.45
5	1:12	20	70	1191.95 ± 9.98
6	1:12	30	0	912.07 ± 93.34
7	1:16	10	70	1636.80 ± 79.20
8	1:16	20	0	1048.09 ± 86.52
9	1:16	30	35	1652.33 ± 1.33

* Gallic acid equivalents mg/100 g of dry material.

**Table 2:** Analysis of variance.

Effect	SS	Df	MS	F	*p*	Contribution (%)
Solid/liquid ratio	367,362.70	2	183,681.40	3.39	0.23	54.09
Time	87,467.16	2	43,733.58	0.81	0.55	12.88
Ethanol concentration	116,086.30	2	58,043.14	1.07	0.48	17.09
Error	108,233.50	2	54,116.77			15.94
Total	679,149.66	8				100.00

SS = Standard deviation; df = degrees of freedom; MS = Mean square; F = Test F; *p* = *p*-value.

**Table 3:** Optimal extraction conditions.

Factor	Level	Value	Effect Size	Standard Error
Solid/liquid ratio	3	1:16	275.04	134.31
Time (min)	1	10	131.81	134.31
Ethanol concentration (%)	2	35	97.69	134.31
Expected			1675.23	
Experimental validation (GAE mg/100 g)			1689.98 ± 86.43	

**Table 4:** Quantitative characterization of the phenolic content and antioxidant activities of the purified polyphenolic fraction of *H. patens*.

Test	Result
Total polyphenols (GAE mg/100 g *)	3552.84 ± 7.25
Flavonoids (CatE mg/100 g *)	1316.17 ± 0.27
Condensed tannins (PC-B1E mg/100 g *)	1694.87 ± 22.21
Redox potential (mV)	553.93 ± 1.22
DPPH reduction (%)	63.08 ± 0.42
Half-maximal inhibitory concentration (IC50) (mg/L)	67.49
Lipid oxidation inhibition (%)	77.78 ± 2.78

* Data are expressed per 100 g of purified polyphenol fraction; GAE: Gallic acid equivalents; CatE: Catechin equivalents; PC-B1E: Procyanidin B1 equivalent.

**Table 5:** Inhibitory effect of purified polyphenol fraction of *H. patens* against Gram-negative and Gram-positive bacteria.

Strain	Control Inhibition * (mm)	*H. patens* Polyphenols Inhibition ** (mm)	*H. patens* Polyphenols Inhibition *** (%)	Minimum Inhibitory Concentration (μg/mL)
Gram-negative bacteria				
*P. aureuginosa ATCC 10145*	30.00	0.00	0.00	-
*E. cloacae (Clinical Isolate)*	0.00	0.00	0.00	-
*E. coli ATCC 15597*	25.00	0.00	0.00	-
*E. coli β (-)*	21.00	19.00	90.50	94.00
*K. pneumoniae*	12.00	9.00	75.00	250.00
Gram-positive bacteria				
*S. aureus ATCC 29213*	0.00	0.00	0.00	-
*S. epidermidis*	20.00	0.00	0.00	-
*E. faecalis ATCC 19433*	25.00	20.00	80.00	125.00

* Ciprofloxacin 100 µg/mL in DMSO 70%. ** Purified phytochemicals fraction from *H. patens* 1000 µg/mL in DMSO 70%. *** Compared against positive control.

**Table 6:** HPLC-ESI-MS identification of phytochemicals extracted from *H. patens*.

Peak No.	Retention Time (min)	UV λ_máx_ (nm)	M.W.	[M-H]^−^ *m*/*z*	Fragments (*m*/*z*)^2^	Tentative Identification	Molecular Formula	Phenolic Group	References
1	15.90	281, 329	354.31	352.9	**191.05**, 192.03, 179.10, 173.10	Scopoletin-7-*O*-glucoside isomer	C_16_H_18_O_9_	Coumarin	[22]
2	18.44	281, 329	354.31	352.9	**191.05**, 192.03, 179.10, 173.10	Scopoletin-7-*O*-glucoside isomer	C_16_H_18_O_9_	Coumarin	[22]
3	19.78	281	578.5	576.9	**407.04**, 424.98, 451.12, 289.12, 299.10	(E)Cat–(E)Cat (Epicatechin)	C_30_H_26_O_12_	Proanthocyanidin	[23]
4	21.38	281	290.26	288.9	**245.96**, 255.94, 256.98, 227.02, 229, 266.92, 241.03, 213.07, 239.03, 199, 163.02, 197.05, 240.00, 228.10	(Epi) Catechin	C_15_H_14_O_6_	Flavonoid	[24]
5	25.27	258, 360	594.5	593.0	**353.12**, 383.01	Apigenin-6,8-*C*-di-glucoside (Vicenin II)	C_27_H_30_O_15_	Flavonoid	[25]
6	27.81	266, 351	610.5	609.0	**301.11**, 300.15, 302.13, 271.09, 255.10, 343.10	Quercetin-deoxyhexosyl-hexoside	C_27_H_30_O_16_	Flavonoid	[23]
7	29.35	268, 352	594.5	593.0	**383.01**, 353.12	Apigenin 6,8-di C-glucoside (Vicenin II isomer)	C_27_H_30_O_15_	Flavonoid	[26]
8	30.37	245, 333	594.5	593.0	**285.1**, 284.12, 286.09, 255.12, 327.11, 257.13, 256.14	Kaempferol-3-*O*-rutinoside	C_27_H_30_O_15_	Flavonoid	[27]
9	31.67	239	396	395.0	**363.05**, 380.10, 319.11, 381.01, 364.08, 325.15, 320.10, 211.95	Unknown	-		[28,29]
10	36.14	239	342.30	340.9	**216.98**, 219.01, 230.98, 179.03, 177.02, 218.00, 296.97, 322.88, 191.05, 280.96, 281.97, 190.07, 220.05, 216.10, 231.95, 203.00	Caffeic acid-*O*-glucoside	C_15_H_18_O_9_	Hydroxycinnamic acid	[30]

**Table 7:** Factors considered for the optimization of extraction conditions.

No.	Factor	Level 1	Level 2	Level 3
1	Solid/liquid ratio (*w/v*)	1:8	1:12	1:16
2	Extraction time (min)	10	20	30
3	Ethanol concentration (%)	0	35	70

## Data Availability

Not applicable.

## Supplementary Materials

The following supporting information can be downloaded at: https://www.mdpi.com/article/10.3390/molecules27248845/s1, Figure S1. Mass spectrum of identified compounds. (A,B) are Scopoletin-7-*O*-glucoside isomer, (C,E) Cat–(E)Cat (Epicatechin), (D) (Epi) Catechin, (E,G) Apigenin-6,8-*C*-di-glucoside (Vicenin II), (F) Quercetin-deoxyhexosyl-hexoside, (H) Kaempferol-3-*O*-rutinoside, (I) Unknown, (J) Caffeic acid-*O*-glucoside.
