# Supplementary material for: The Ultrasound-Assisted Extraction of Polyphenols from Mexican Firecracker (Hamelia patens Jacq.): Evaluation of Bioactivities and Identification of Phytochemicals by HPLC-ESI-MS
Source: Molecules. 2022 Dec 13;27(24):8845. doi: 10.3390/molecules27248845 (PMC9785907; doi:10.3390/molecules27248845)
Supplement: Supplementary file 1 [file molecules-27-08845-s001.zip › molecules-2046839-supplementary.pdf]

A)

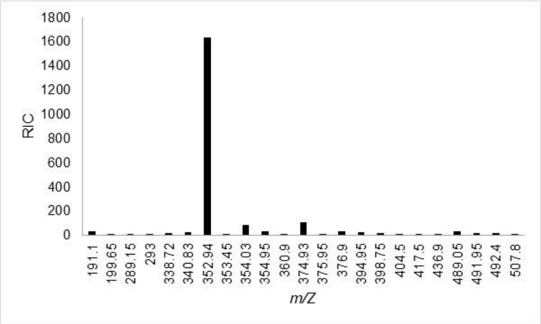
RIC
1800
1600
1400
1200
1000
800
600
400
200
0
191.1 199.65 289.15 293 338.72 340.83 352.94 353.45 354.03 354.95 360.9 374.93 375.95 376.9 394.95 398.75 404.5 417.5 436.9 489.05 491.95 492.4 507.8
m/Z

B)

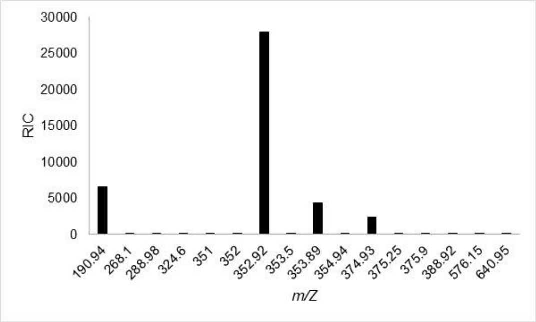
RIC
30000
25000
20000
15000
10000
5000
0
190.94 268.1 288.98 324.6 351 352 352.92 353.5 353.89 354.94 374.93 375.25 375.9 388.92 576.15 640.95
m/Z

C)

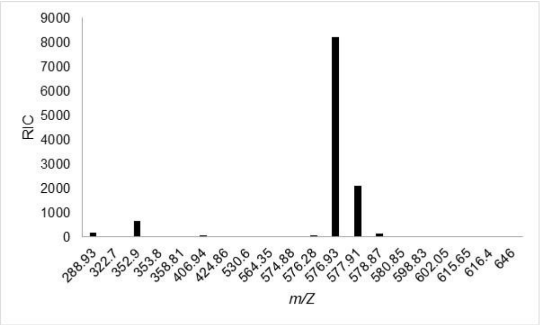
RIC
9000
8000
7000
6000
5000
4000
3000
2000
1000
0
288.93 322.7 352.9 353.8 358.81 406.94 424.86 530.6 564.35 574.88 576.28 576.93 577.91 578.87 580.85 598.83 602.05 615.65 616.4 646
m/Z

D)

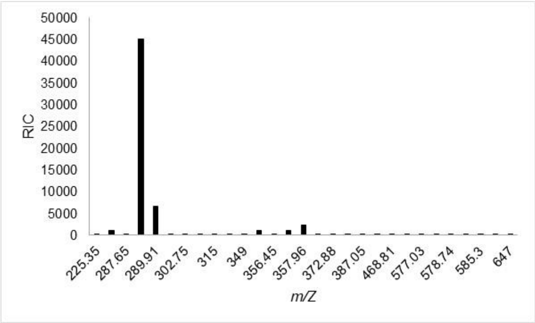
RIC
50000
45000
40000
35000
30000
25000
20000
15000
10000
5000
0
225.35 287.65 289.91 302.75 315 349 356.45 357.96 372.88 387.05 468.81 577.03 578.74 585.3 647
m/Z

E)

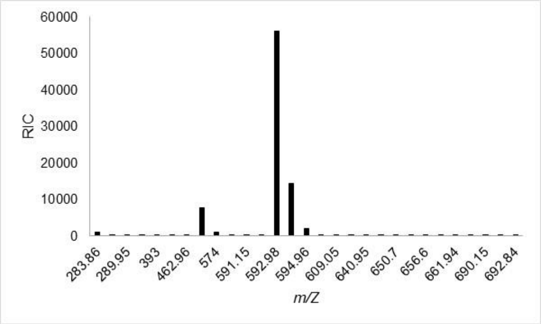
RIC
60000
50000
40000
30000
20000
10000
0
283.86 289.95 393 462.96 574 591.15 592.98 594.96 609.05 640.95 650.7 656.6 661.94 690.15 692.84
m/Z

F)

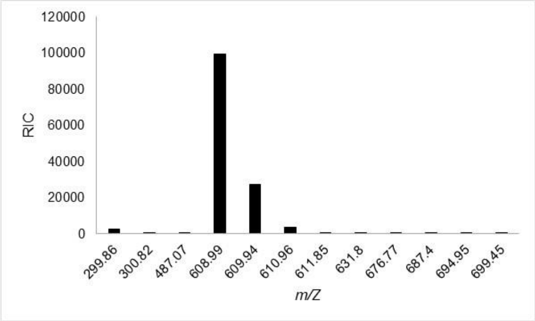
RIC
120000
100000
80000
60000
40000
20000
0
299.86 300.82 487.07 608.99 609.94 610.96 611.85 631.8 676.77 687.4 694.95 699.45
m/Z

G)

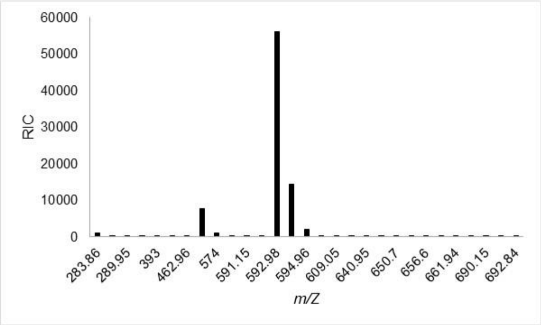
RIC
60000
50000
40000
30000
20000
10000
0
283.86 289.95 393 462.96 574 591.15 592.98 594.96 609.05 640.95 650.7 656.6 661.94 690.15 692.84
m/Z

H)

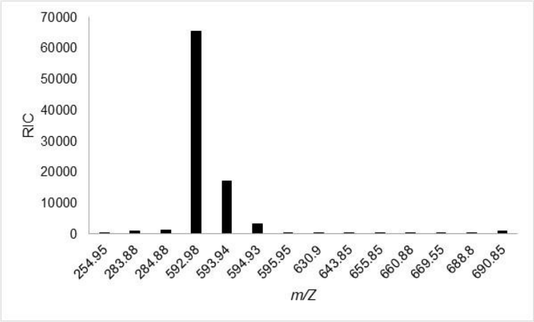
RIC
70000
60000
50000
40000
30000
20000
10000
0
254.95 283.88 284.88 592.98 593.94 594.93 595.95 630.9 643.85 655.85 660.88 669.55 688.8 690.85
m/Z

I)

J)

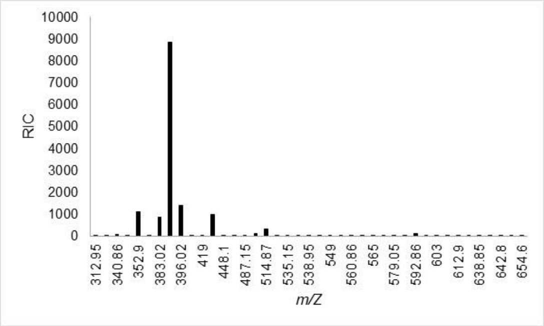

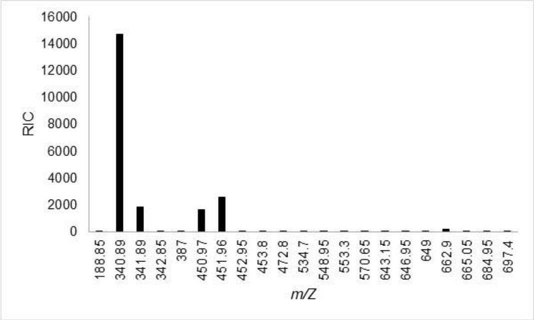

Figure S1. Mass spectrum of identified compounds. (A,B) are Scopoletin-7-*O*-glucoside isomer, (C,E) Cat–(E)Cat (Epicatechin), (D) (Epi) Catechin, (E,G) Apigenin-6,8-*C*-di-glucoside (Vicenin II), (F) Quercetin-deoxyhexosyl-hexoside, (H) Kaempferol-3-*O*-rutinoside, (I) Unknown, (J) Caffeic acid-*O*-glucoside.
